# Downregulation of RPL6 by siRNA Inhibits Proliferation and Cell Cycle Progression of Human Gastric Cancer Cell Lines

**DOI:** 10.1371/journal.pone.0026401

**Published:** 2011-10-17

**Authors:** Qiong Wu, Yawen Gou, Qiaomin Wang, Haifeng Jin, Lina Cui, Yongguo Zhang, Lijie He, Jingbo Wang, Yongzhan Nie, Yongquan Shi, Daiming Fan

**Affiliations:** 1 State Key Laboratory of Cancer Biology, Xijing Hospital of Digestive Diseases, the Fourth Military Medical University, Xi'an, Shaanxi, China; 2 Department of Digestive Diseases, The Affiliated Provincial Hospital of Anhui Medical University, Hefei, Anhui Province, China; The University of Hong Kong, China

## Abstract

Our previous study revealed that human ribosomal protein L6 (RPL6) was up-regulated in multidrug-resistant gastric cancer cells and over-expression of RPL6 could protect gastric cancer from drug-induced apoptosis. It was further demonstrated that up-regulation of RPL6 accelerated growth and enhanced in vitro colony forming ability of GES cells while down-regulation of RPL6 exhibited the opposite results. The present study was designed to investigate the potential role of RPL6 in therapy of gastric cancer for clinic. The expression of RPL6 and cyclin E in gastric cancer tissues and normal gastric mucosa was evaluated by immunohistochemisty. It was found that RPL6 and cyclin E were expressed at a higher level in gastric cancer tissues than that in normal gastric mucosa and the two were correlative in gastric cancer. Survival time of postoperative patients was analyzed by Kaplan- Meier analysis and it was found that patients with RPL6 positive expression showed shorter survival time than patients that with RPL6 negative expression. RPL6 was then genetically down-regulated in gastric cancer SGC7901 and AGS cell lines by siRNA. It was demonstrated that down-regulation of RPL6 reduced colony forming ability of gastric cancer cells in vitro and reduced cell growth in vivo. Moreover, down-regulation of RPL6 could suppress G1 to S phase transition in these cells. Further, we evidenced that RPL6 siRNA down-regulated cyclin E expression in SGC7901 and AGS cells. Taken together, these data suggested that RPL6 was over-expressed in human gastric tissues and caused poor prognosis. Down-regulation of RPL6 could suppress cell growth and cell cycle progression at least through down-regulating cyclin E and which might be used as a novel approach to gastric cancer therapy.

## Introduction

Gastric cancer, with high morality, was one of the most malignant cancers in Asian countries. Its occurrence was a process with multi-factors and multi-steps, involved in alterations in many molecules, including activation of proto-oncogenes, inactivation of tumor-suppressor genes, alterations in cell cycle related proteins and so on. In the past decade, a great number of proto-oncogenes and tumor-suppressor genes have been found. In spite of the sizable number of genes already described, new genes with oncogenic potential or tumor-suppressing activities are still being identified. However, the molecular mechanism of gastric carcinogenesis remains unclear.

Ribosome is essential for protein synthesis in all cells, which is constituted of ribosomal RNAs (rRNAs) and ribosomal proteins (RPs). Many RPs also play various roles that are independent of protein biosynthesis, which is called extra-ribosomal function [1]. Increasing more research demonstrated that disorder of protein translation participated in tumorigenesis and many ribosomal proteins were dysfunctional in tumors, but the mechanisms were still unknown [2]. It has been proposed that many RPs act as haploinsufficient tumor suppressors in fish and many RPs genes might also be oncogenes in human kind [3]. The earliest report on relationships between RPs and tumors was published in 90 s of the 20^th^ century, which demonstrated the association of RPS19 with colon carcinoma progression and differentiation [4].Thereafter, more and more RPs were found dysfunctional in tumors, such as high expression of RPL19 in breast tumors [5], over-expression of RPL7a and RPL37 in prostate-cancer tissue samples [6], higher expression of RPL15 in esophageal cancer [7], RPs as early detection marker of human squamous cell carcinoma of the uterine cervix [8], and over-expression of RPL36a associated with cellular proliferation in hepatocellular carcinoma [9] and so on. Reports regarding the association of RPs with carcinogenesis were still increasing [2], [10]. However, the exact roles of RPs in tumor development are diverse and still need further clarification.

Previously, we analyzed the mRNA profiles of a human gastric cancer cell line SGC7901 and its multidrug-resistant variant SGC7901/VCR by differential displayed PCR and suppression subtractive hybridization [11], [12]. RPL6 was identified as one of the over-expressed genes in SGC7901/ADR compared to SGC7901, which was further confirmed by RT-PCR and Northern blot analysis [13]. Subsequent studies revealed that up-regulation of RPL6 protected gastric cancer cells from adriamycin-induced apoptosis and enhanced the resistance of gastric cancer cells to multiple chemotherapeutic drugs, including vincristine, adriamycin, etopside, cisplatin and 5-fludrouracil [13]. These data indicated that RPL6 could promote the malignant phenotypes of gastric cancer cells, which led us to conduct the subsequent study to further explore the exact role of RPL6 in carcinogenesis and development of gastric cancer. Then we discovered that genetically up-regulation of RPL6 in GES cells could accelerate the growth and enhance in vitro colony forming ability of GES cells while down-regulation of RPL6 could exhibit the opposite results. Moreover, up-regulation of RPL6 could promote G1 to S phase transition of GES cells. Further study demonstrated that RPL6 up-regulated cyclin E expression in GES cells [14]. Taken together, these data suggested that RPL6 could promote the malignant phenotypes of gastric cancer cells and RPL6 might play an oncogenic role in the tumorigenesis and development of gastric cancer, which led us to conduct the present study to explore the potential role of RPL6 in gene therapy of gastric cancer and might be used as a novel approach to gastric cancer therapy.

## Results

### RPL6 and cyclin E expression in human gastric cancer specimens and matched adjacent non-neoplastic tissues

**Figure 1:**
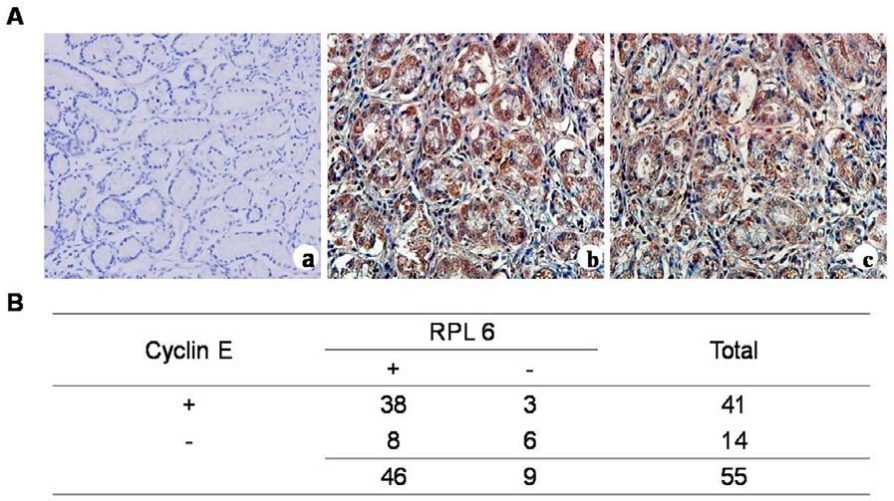
RPL6 and cyclinE expression in human gastric cancer specimens and matched adjacent non-neoplastic tissues. **A.** Representative images shown are negative immunohistochemical control of IgG (a) and positive immunohistochemical staining of RPL6 (b) and cyclin E (c) in the same gastric cancer tissues (magnification ×200). **B.** Statistical analysis of data on the correlation between RPL6 and cyclinE expression in human gastric cancer specimens. (p<0.05).

**Figure 2:**
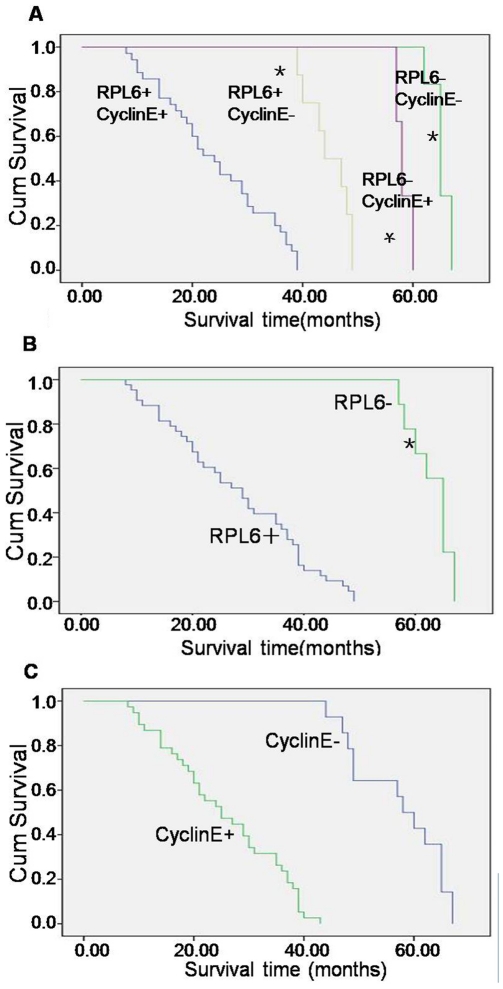
Upregulation of RPL6 and Cyclin E expression shorten patients' survival time after followed up. **A.** Patients with both RPL6 and Cyclin E positive, RPL6 positive and Cyclin E negative, RPL6 negative and Cyclin E positive, both RPL6 and Cyclin E negative. **B.** Patients with RPL6 positive and negative. **C.** Patients with Cyclin E positive and negative. p<0.01 vs both RPL6 and Cyclin E positive and the corresponding control; * p<0.01 vs RPL6 positive and RPL6 negative.

To explore the relationships between RPL6 and cyclin E expression with the development of gastric cancer, we detected RPL6 and cyclin E expression in human gastric cancer tissues and matched adjacent non-neoplastic tissues by immunohistochemical staining. The results revealed that RPL6 and cyclinE localized almost in cytoplasm and only occasionally in nuclei in gastric cancer cells (Fig. 1A). Further analysis discovered that the positive ratio (including specimens that stained) of RPL6 was 84% (46 of 55 patients) while the positive ratio of cyclin E was 75% (41 of 55 patients) in human gastric cancer specimens. The positive ratio of both RPL6 and cyclin E was 70% (38 of 55 patients) while the negative ratio (including specimens that did not stain) of both RPL6 and cyclin E was 11% (6 of 55 patients) in human gastric cancer specimens. The positive ratio of both RPL6 and cyclin E was 18% (8 of 55 patients) in matched adjacent non-neoplastic tissues (data not shown). As for the differences between RPL6 expression and cyclinE expression in human gastric cancer specimens, there was no difference. The present study demonstrated that RPL6 and cyclin E expressions in human gastric cancer specimens were relative (Fig. 1B, p<0.05), which may play a con-generous role in the development of gastric cancer and RPL6 and cyclin E may serve as a biomarker for gastric cancer diagnosis and a gene target for gastric cancer therapy. At the end of follow up, survival time of the patients were analyzed by Kaplan- Meier method and the results revealed that during patients that were followed up, patients with both RPL6 positive and cyclin E positive showed a shorter survival time and poorer prognosis than those with both RPL6 negative and cyclin E negative expression, or those with RPL6 positive and cyclin E negative or with RPL6 negative and cyclinE positive expression respectively (Fig. 2 A, p<0.01), which can be revealed that patients with both RPL6 positive and cyclin E positive showed poor prognosis. It is also demonstrated that patients with RPL6 negative expression showed longer survival time than RPL6 positive ones (Fig. 2B, p<0.01), and patients with cyclin E positive expression showed poor prognosis (Fig. 2C, p<0.01). Therefore, a conclusion can be drawn that RPL6 may served as a biomarker for evaluating the prognosis of gastric cancer patients.

### Expression of RPL6 in gastric cancer cell lines and its derivates

**Figure 3:**
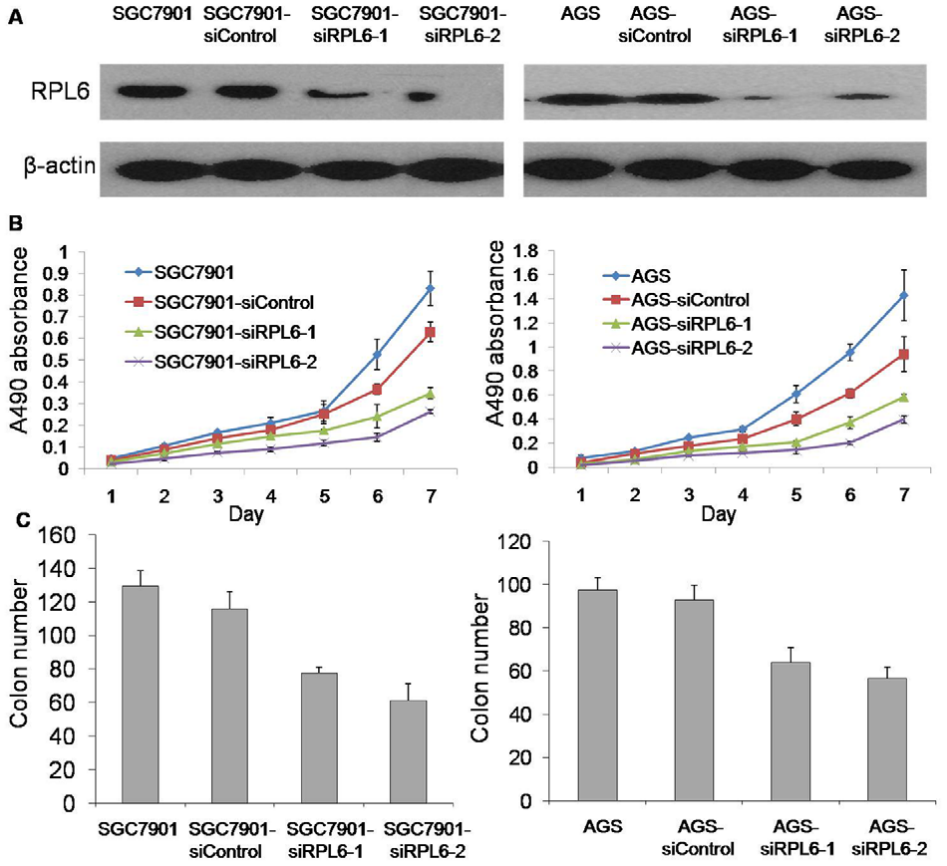
Downregulation of RPL6 represses gastric cancer cell growth in vitro. **A.** Detection of RPL6 in SGC7901/AGS, SGC7901/AGS-siRPL6-1,SGC7901/AGS-siRPL6-2, SGC7901/AGS-siRPL6-Control cells. β-actin was detected as a loading control. **B.** The growth curves of SGC7901/AGS cell variants with down-regulated RPL6 were determined. The proliferation of SGC7901/AGS cell derivates was monitored with MTT assay as described in “Materials and Methods” and their growth curves were plotted. Error bars correspond to mean ± SD. P<0.05 compared with control cells. **C.** Colony formation ability of SGC7901/AGS cell variants with down-regulated RPL6 was determined with soft agar colony formation assay as described in “Materials and Methods”. Values represent the mean ± SD of triplicates in each experiment. Shown are representative of at least three separate experiments. (p<0.05).

To further explore the potential role of RPL6 in gene therapy of gastric cancer, SGC7901 and AGS cells were transfected with RPL6-specific siRNA and the mixed pool of G418-resistant cell variants was produced. The G418-resistant variants harboring RPL6-specific siRNA were accordingly named as SGC7901-siRPL6-1, AGS-siRPL6-1 and SGC7901-siRPL6-2, AGS-siRPL6-2. The G418-resistant cells harboring scramble siRNA plasmids were designed as SGC7901-siControl and AGS-siControl respectively. As shown in Figure 3A, RPL6 expression was significantly down-regulated in SGC7901-siRPL6-1, AGS-siRPL6-1 and SGC7901-siRPL6-2, AGS-siRPL6-2 cells compared with parental cells and the control cells. In the present study, SGC7901-siRPL6-2 and AGS-siRPL6-2 showed more effective inhibitory results, which indicated that the SGC7901-siRPL6-2 and AGS-siRPL6-2 cells could be used as a good cell model to clarify the potential role of RPL6 in gene therapy of gastric cancer for clinic.

### Downregulation of RPL6 suppress gastric cancer cell growth in vitro

To study whether down-regulation of RPL6 expression could inhibit the growth of SGC7901 and AGS cells, parental cells, control cells and their variants were seeded into culture plates and their growth was monitored everyday with MTT assay. As indicated by the growth curves in Figure 3B, control (SGC7901-siControl and AGS-siControl) cells showed a similar growth rate to parental (SGC7901 and AGS) cells respectively, whereas SGC7901-siRPL6-1, AGS-siRPL6-1 and SGC7901-siRPL6-2, AGS-siRPL6-2 cells exhibited lower growth rate than Control cells, and SGC7901-siRPL6-2, AGS-siRPL6-2 cells showed the lowest growth rate (Fig. 3B, p<0.05). The anchorage-independent growth of these cells was further analyzed with soft agar colony formation assay. It was revealed that SGC7901-siRPL6-1, AGS-siRPL6-1 and SGC7901-siRPL6-2, AGS-siRPL6-2 cells produced much less cell colonies in soft agar than their parental cells and control cells (Fig. 3C, p<0.05), cells named with SGC7901-siRPL6-2 and AGS-siRPL6-2 produced the least colonies. These data suggested that down-regulation of RPL6 could suppress gastric cancer cell growth and inhibit the colony formation ability of SGC7901 and AGS cells.

### RPL6 siRNA inhibited tumorigenesis of gastric cancer cells in vivo

**Figure 4:**
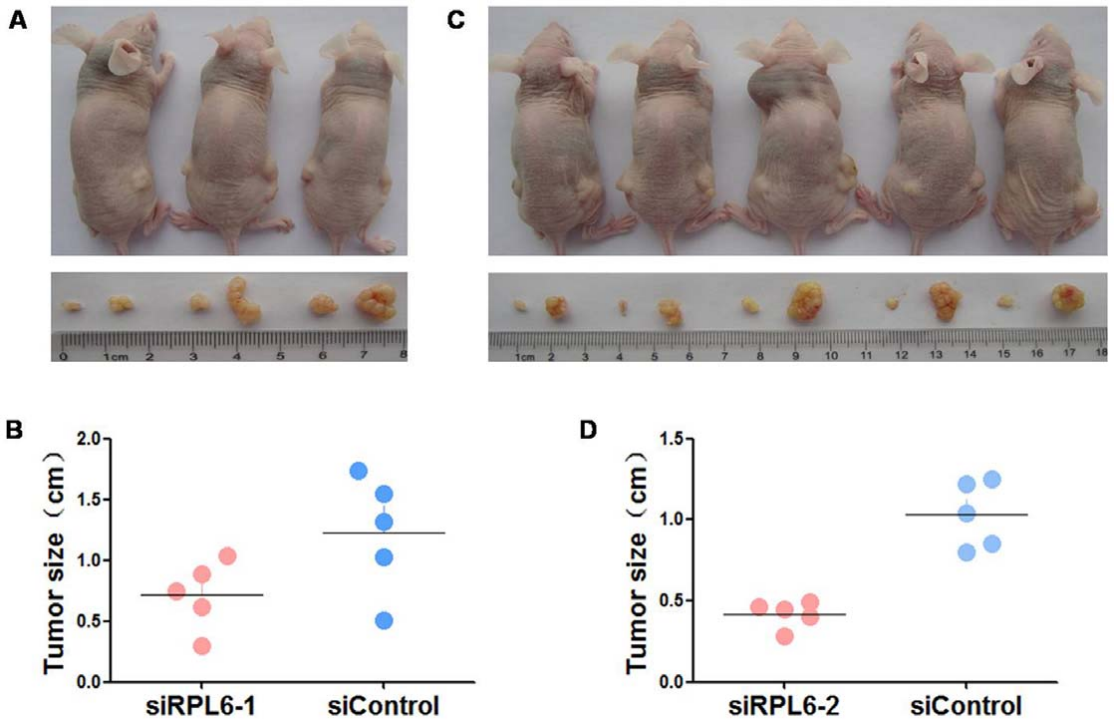
Down-regulation of RPL6 inhibits tumorigenesis in vivo. **A.** SGC7901 cells stably transfected with RPL6-specific siRNA1 (left) or with RPL6-scramble siRNA were injected into nude mice. **B.** Four weeks after the injection, mice were photographed and killed. Tumor sizes were measured. Error bars indicate mean ± SD. p<0.05 compared with the control group. **C.** SGC7901 cells stably transfected with RPL6-specific siRNA2 (left) or with RPL6-scramble siRNA were injected into nude mice. **D.** Four weeks after the injection, mice were photographed and killed. Tumor sizes were measured. Error bars indicate mean ± SD. p<0.05 compared with the control group.

To further confirm the effects of RPL6 on tumorigenesis of gastric cancer, tumor formation assay was performed in nude mice. SGC7901, AGS and SGC7901-siRPL-2, AGS-siRPL6-2 cells were subcutaneously inoculated into the right or left upper back of athymic nude mice at a single site. (Fig. 4A and C) Four weeks later, mice were killed and the transplanted tumors were excised and tumor sizes were evaluated (Fig. 4B and D, p<0.05). There was a significant decrease of sizes of xenografts in RPL6 down-regulated cells. These data indicated that knock-down of RPL6 could suppress gastric cancer cell tumorigenesis in vivo and RPL6 may play a key role in promoting the malignant growth of gastric cancer cells in vivo.

### RPL6 decelerated G1-S phase transition of SGC7901 cells

**Figure 5:**
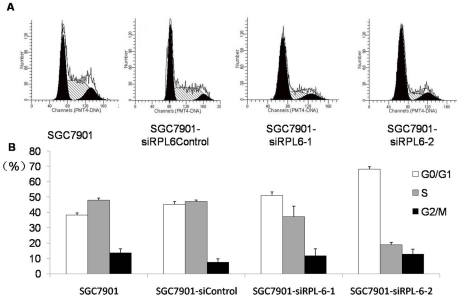
Cell cycle analysis of SGC7901 cells and its derivates. **A.** SGC7901 and its derivates in log phase were harvested and subjected to cell cycle analysis. The cell cycle phase distribution was evaluated by FACS. Shown are representative images of four separate experiments. **B.** Cell cycle distribution of SGC7901, SGC7901-siRPL6-1, SGC7901-siRPL6-2 and SGC7901-siRPL6Control cell lines. The cell cycle distribution was calculated and expressed as mean ± SD of four separate experiments. p<0.05.

To uncover the mechanisms underlying the suppressive role of RPL6 in gastric cancer cell growth, the effects of RPL6 on cell cycle distribution of SGC7901 cells were analyzed (Fig. 5A). As shown in Figure 5B, SGC7901-siRPL6-1 cells showed slightly increased percentage of G1 phase and decreased percentage of S phase, while SGC7901-siRPL6-2 cells displayed significantly increased percentage of G1 phase and decreased percentage of S phase (P<0.05) compared with parental cells and Control cells. These indicated that RPL6-specific siRNA could decelerate the G1-S transition of SGC7901 cells.

### RPL6 down-regulated expression of cyclin E in gastric cancer cells at the protein level

**Figure 6:**
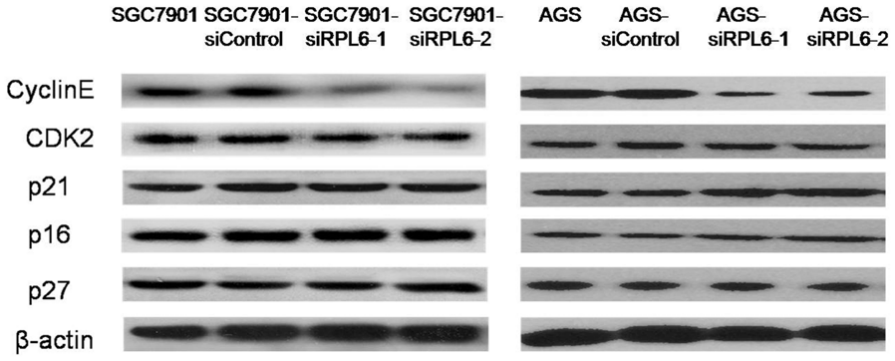
Detection of cell cycle-related proteins in SGC7901 cells. The expression of cyclin E, CDK2, p21, p16 and p27 in SGC7901 and AGS cell variants with down-regulated RPL6 was determined by Western blot using the corresponding antibodies. β-actin was detected as a loading control.

It is well known that G1-S progression is controlled by cyclin D/CDK4 and cyclin E/CDK2 complexes, which are regulated by INK4 family members, p21 and p27, respectively. The expression of these regulators in SGC7901 and AGS cells and its variants were determined by Western Blot analysis. As indicated in Figure 6, expression of cyclin E was significantly down-regulated in SGC7901-siRPL6-2 and AGS-siRPL6-2 cells. Expressions of CDK2, p16, p21 and p27 were unchanged in SGC7901 and AGS cell variants. These data suggested that down-regulation of RPL6 decreased the expression of cyclin E in SGC7901 and AGS cells, which might delineate the potential role of RPL6 and cyclin E in the development of gastric cancer and RPL6 may used as a gene target for gastric cancer in the clinic.

## Discussion

The present study demonstrated that RPL6 and cyclin E were over-expressed in gastric cancer tissues and there is a positive correlation between them in gastric cancer tissues. Follow up results revealed that patients with RPL6 negative expression showed longer survival time than those with RPL6 positive expression, which suggested that RPL6 positive patients showed poor prognosis. Further research demonstrated that down-regulation of RPL6 by RPL6-specific siRNA inhibited the proliferation and anchorage-independent growth of gastric cancer cell lines in vitro, repressed cell cycle progression and suppressed cyclin E expression, Moreover, knockdown of RPL6 by RPL6-specific siRNA suppressed tumor formation ability of gastric cancer cells in vivo.

In our previous study, differentially expressed genes associated with the multidrug resistance of gastric cancer cells were isolated using differential display RT-PCR and subtractive hybridization [11], [12]. RPL6, a component of the large subunit of ribosome, was thus isolated as a clone over-expressed in multidrug-resistant gastric cancer cells [11]. It was demonstrated that RPL6 could protect gastric cancer cells from chemotherapeutic drug-induced apoptosis [13]. These data suggested that RPL6 might involve in the regulation of the malignant phenotype of gastric cancer. Further study was designed to explore the role of RPL6 in the tumorigenesis and development of gastric cancer, and results suggested that up-regulation of RPL6 accelerated growth and in vitro colony forming ability of GES cells whereas down-regulation of RPL6 exhibited opposite results. Also, up-regulation of RPL6 accelerated G1 to S phase transition of GES cells at least through up-regulation of cyclin E expression while down-regulation of RPL6 showed opposite results [14]. Taken together, these data suggested that RPL6 might play an oncogenic role in tumorigenesis and development of gastric cancer and might serve as a novel approach to gene therapy of gastric cancer.

Nowadays, increasing more patients died of cancer because of the malignant phenotypes of them. Cancer therapy disturbed people worldwide. Besides chemotherapy and surgery treatment, gene therapy is a novel and promising cancer treatment. One of the most commonly used methods for gene therapy is RNA interference (RNAi). RNAi, a highly conserved gene silencing mechanism plays an important role in the regulation of gene expression. Gene silencing by a post-transcriptional mechanism involving homologous double-stranded RNA was first discovered in artificial systems where double strand RNA (dsRNA) was introduced through injection or by expression of transgenic constructs. This phenomenon occurs by small interfering RNA (siRNA) in the cytoplasm of mammalian cells. In siRNA technology, two delivery mechanisms were considered, direct delivery of synthetic siRNA nucleotide and introduction of a plasmid DNA (pDNA) encoding a short hairpin construct (shRNA) that would be enzymatically degraded into siRNA [15]. SiRNA played its inhibitory roles by matching with the sequences of mRNA and inhibited the consequent mRNA expression and translation, and worked as a negative regulation. Nowadays, more and more scientists have discovered that knocked down gene expression by siRNA could significantly suppress tumor growth and metastasis. X-linked inhibitor of apoptosis protein (XIAP), an important member of the inhibitors of apoptosis protein (IAP) family, is involved in control of cell division, cell proliferation and inhibition of apoptosis [16], [17]. Down-regulation of XIAP by siRNA could significantly suppress proliferation of tumor cells and sensitize tumor cells to chemotherapeutic agents [18], [19]. Osteopontin (OPN) protein, which was over-expressed in the majority of gastric cancer patients and associated with its pathogenesis, played an important role in the proliferation, invasion, metastasis and survival of gastric cancer [20]. Then down-regulation of OPN by OPN-specific siRNA significantly inhibited the growth, migration and invasion of gastric cancer cells [21]. CML66, a novel tumor antigen which played an oncogenic role, was over-expressed in a majority of cancer cell lines and cancers [22], knocking down its expression by CMl66-specific siRNA significantly inhibited proliferation, invasion and metastasis of HeLa cells both in vivo and in vitro experiments [23]. FABP5, which encodes cutaneous fatty acid binding protein (C-FABP), is up-regulated in prostate cancer and acts as a putative oncogene. Down-regulation of FABP5 by FABP5-specific siRNA effectively inhibited prostate cancer cell growth in nude mice [24]. Moreover, when ribosomal protein RPS13 which enhanced gastric cancer cell line SGC7901 growth was knocked down by RPS13-specific siRNA, it significantly inhibited SGC7901 cell growth and colony forming ability in vitro and suppressed tumor formation ability in nude mouse [25]. Previous research also found that systemic delivery of siRNA via atelocollagen, which specifically targets tumors, is safe and feasible for cancer therapy in prostate cancer [26]. Therefore, evidence above suggested that siRNA, by inhibiting gene expression could be a novel approach to cancer therapy and make gene therapy of cancer feasible for clinical treatment.

In the present study, expressions of RPL6 and cyclin E in gastric cancer tissues and the matched adjacent non-neoplastic tissues were detected and the results indicated that RPL6 and cyclin E showed higher expression in gastric cancer tissues than in matched adjacent non-neoplastic tissues. Furthermore, there is a correlation between RPL6 and cyclin E expression in gastric cancer tissues, which suggested that they may play a con-generous role in the development of gastric cancer and RPL6 might serve as a biomarker for gastric cancer diagnosis. Follow up results demonstrated that patients with both RPL6 positive and cyclin E positive expressions showed a shorter survival time and poorer prognosis than the corresponding control and also indicated that patients with RPL6 negative expression showed longer survival time and better prognosis than patients with RPL6 positive expression, which suggested that RPL6 may served as a biomarker for gastric cancer prognosis and a gene target for gastric cancer therapy in clinic.

To further explore the potential feasibility of RPL6 as gene therapy target of gastric cancer in clinic, we knocked down RPL6 expression in SGC7901 and AGS cells by RPL6-specific siRNA, results showed that down-regulation of RPL6 in gastric cancer parental cell lines (SGC7901 and AGS) effectively inhibited cell growth and in vitro colony forming ability. Further, cell cycle analysis indicated that down-regulation of RPL6 decelerated G1 to S phase of gastric cancer cells. Western blot analysis demonstrated that RPL6 inhibited cell cycle progression through down-regulation of cyclin E. Tumor formation experiments in nude mice suggested that down-regulation of RPL6 could suppress tumor formation in vivo.

Cyclin E, a key cell cycle protein that played key roles in G1-S transition was in disorder in many different tumors [27], [28], [29], [30], [31]. Previous research demonstrated that cyclin E was an important molecule in tumorigenesis. In the present study, we discovered that cyclin E expression, together with RPL6 was higher in human gastric cancer tissues than that in matched adjacent non-neoplastic tissues, which suggested the role of cyclin E played in the development of gastric cancer. Further research demonstrated that RPL6 affected cell growth by regulating cyclin E, which showed that high expression of cyclin E accelerated cell growth while low expression of cyclin E decelerated cell proliferation. Cyclin E and RPL6 may play con-generous roles in the development of gastric cancer.

In conclusion, the present study demonstrated that RPL6 and cyclinE expression were associated with each other in gastric cancer tissues and they might play con-generous roles in the development of gastric cancer, which suggested that RPL6 may used as a diagnostic tool in gastric cancer. Follow up data analysis demonstrated that patients with RPL6 negative expression showed longer survival time and better prognosis than patients with RPL6 positive expression postoperatively, which suggested that RPL6 served as a biomarker for gastric cancer prognosis and a novel gene target for gastric cancer therapy. Further study indicated that down-regulation of RPL6 expression by RPL6-specific siRNA inhibited SGC7901 cell growth and in vitro colony formation ability, decelerated SGC7901 cell G1 to S phase transition, and suppressed tumor formation in vivo. In conclusion, our present study confirmed that RPL6 siRNAs might be used as a novel approach to gastric cancer therapy for clinic.

## Materials and Methods

### Ethics Statement

For tissue specimens, all patients were informed consent to use excess pathological specimens for research purposes. The protocols used in this study were approved by the hospital's Protection of Human Subjects Committee. The use of human tissues was approved by the institutional review board of the Fourth Military Medical University and was conformed to the Helsinki Declaration, and to local legislation. Patients offering samples for the study signed informed consent forms. For animal research, all procedures for animal experimentation were performed in accordance with the Institutional Animal Care and Use Committee guidelines of the Experiment Animal center of the Fourth Military Medical University. The approval ID for using the animals was No. 10218 by Experiment Animal Center of the Fourth Military Medical University.

### Tissue specimens and cell lines

Tissue specimens embedded with paraffin were collected from 55 patients with gastric cancer who underwent gastrectomy in our hospital between 2004 and 2009. All cases of gastric cancer and adjacent non-tumor tissues were diagnosed clinically and pathologically. Data on clinicopathological features and prognoses of the patients were collected and analyzed retrospectively. A total of 55 patients were followed up until the end of the year 2009 and two of them were lost during the follow up period. Human gastric adenocarcinoma cell line SGC7901 was obtained from the Academy of Military Medical Science, AGS was obtained from the Shanghai Cell Bank (Shanghai, China). The two cell lines were preserved in our institute. All the cell lines were maintained in RPMI1640 (Invitrogen, Carlsbad, CA) supplemented with 10% heat-inactivated fetal calf serum (FCS) at 37°C with 5% CO_2_ in a humidified incubator (Forma Scientific, Marietta, OH).

### Immunohistochemistry and Immunohistochemical scoring

Deparaffinized and rehydrated sections were washed in fresh water for 10 minutes. Heat-induced antigen retrieval was performed for 20 minutes at 95°C with 10 mM citrate sodium buffer (PH 6.0). After the slides were cooled at room temperature for 40 minutes, they were blocked in 3% hydrogen peroxide for 20 minutes and then in normal goat serum confining liquid for 40 minutes. After this, they were allowed to react over night at 4°C with primary antibodies for IgG, RPL6 and cyclinE (Santa Cruz). After rewarming for 40 minutes, they were then reacted with second antibodies (Zhongshan Goldenbridge Biotechnology CO.LTD) for another 40 minutes at room temperature. Then the products were developed with 3,3′-diaminobenzidine and counterstained with haematoxylin. Scoring was completed by a specialist pathologist and a scientist who were blinded to the clinical and pathologic information. In case of discrepancy, a consensus was reached by conferencing. The proportions of positive cells (0 to +3) and the staining intensities (0 to +3) were evaluated separately at a magnification of ×200. The former was calculated by counting the number of stained tumor cells among the total number of tumor cells, for example, when 25% of total cells were stained, the proportion score was +1, and 50% equals to +2, 75% equals to +3, 0% equals to 0. The intensity scoring was evaluated by the color of stained tumor cell nucleus, specifically, score 0 means achromatic, +1 means amber, +2 means yellow, and +3 means brown. Combined scoring +1∼+2 were defined “negative”, and +3∼+6 were considered “positive”.

### Plasmid construction and transfection

Two pairs of hairpin small interfering RNA (siRNA) oligos for RPL6 were designed according to the siRNA design guidelines of TaKaRa Biotechnology Co., Ltd. Compare target sequences to the human genome database in a BLAST search to eliminate from consideration any target sequence with 21 base pairs of homology contiguous to other coding sequences. For oligo-1, sense: 5′-GATCCGGAGAAGGTTCTCGCAACTTTCAAGAGAAGTTGCGAGAACCTTCTCCTTTTTTGGAAA-3′, antisense: 5′AGCTTTTCCAAAAAAGGAGAAGGTTCTCGCAACTTCTCTTGAAAGTTGCGAGAACCTTCTCCG-3′; for oligo-2, sense: 5′-GATCCGTCGAGTTCCTCTACGAAGATTCAAGAGATCTTCGTAGAGGAACTCGATTTTTTGGAAA-3′, antisense: 5′-AGCTTTTCCAAAAAATCGAGTTCCTCTACGAAGATCTCTTGAATCTTCGTAGAGGAACTCGACG-3′; A scramble DNA duplex was also designed as control, for oligo-1, sense: 5′-GATCCGGTGAGATCTTCGACACAGTTCAAGAGACTGTGTCGAAGATCTCACCTTTTTTGGAAA-3′, antisense: 5′-AGCTTTTCCAAAAAAGGTGAGATCTTCGACACAGTCTCTTGAACTG TGTCGAAGATCTCACCG-3′. For annealing to form DNA duplexes, 0.01 M each of sense and antisense oligos were used. The duplexes were diluted and then ligated with pSilencer3.1-H1 neo vector (TaKaRa Biotechnology(Dalian)Co., Ltd). The products were transformed into DH5α-competent cells. Ampicillin-resistant colonies were chosen, identified by restriction digestion, and further confirmed by DNA sequencing. According to the manufacturers' instructions, siRNA plasmids of RPL6 were transfected into SGC7901 cells using Lipofectamine 2000 reagent (Invitrogen, Carlsbad, CA), respectively. Twenty-four hours after transfection, G418 (400 µg/ml) was added into the culture medium for establishing stable clones. The mixed clones were expanded for an additional 2 months. The SGC7901 and AGS cells (also called parental cells) stably expressing exogenic RPL6 were named as SGC7901-siRPL6-1, AGS-siRPL6-1, and SGC7901-siRPL6-2, AGS-siRPL6-2. And cells transfected with siControl were named as SGC7901-siRPL6 Control and AGS-siRPL6 Control (also called control cells).

### Cell proliferation assay

3-(4,5-Dimethylthiazol-2-yl)-2,5-diphenyl-tetrazolium bromide (MTT) assay was performed to evaluate the effect of RPL6 on cell proliferation as previously described [32]. The absorbance at 490 nm (A490) of each well was read on a microplate reader BP800 (Biohit, Helsinki, Finland). Each experiment was performed in quadruplicates and was repeated 3 times.

### Soft agar colony formation assays

Soft agar colony formation assay was used to determine anchorage-independent cell growth potential. Twelve well plates were filled with 0.5 ml of 0.5% noble agar (Invitrogen) in RPMI1640 supplemented with 10% calf serum as a bottom layer and allowed to solidify at 4°C overnight. A total of 200 cells were suspended in 0.25 ml of 0.3% agarose and seeded on the bottom agar. Cell culture plates were maintained for 2 weeks in a CO_2_ incubator. The number of colonies was counted under a microscope.

### Tumorigenicity in nude mice

Tumour formation was carried out to assess the effects of RPL6 on tumorigenicity in vivo. BALB/c nude mice of 4 to 6 weeks were provided by Shanghai Cancer Institute and housed in micro-isolator cages under positive air pressure, and maintained at a constant temperature (22°C) and humidity for the tumorigenicity study. Approximately 3×10^6^ cells at log phase were collected and injected subcutaneously into the upper back of BALB/c nude mice. At least three nude mice were used for each group. The mice were killed 4 weeks later and tumor weight of each mouse was evaluated. All procedures for animal experimentation were performed in accordance with the Institutional Animal Care and Use Committee guidelines of the Experiment Animal center of the Fourth Military Medical University.

### Cell cycle analysis

For analysis of cell cycle phase distribution, SGC7901 and its variants at log phase were harvested and washed twice with ice-cold PBS. Cell pellets were fixed in 70% ethanol, treated with RNase A (Boehringer Mannheim, Indianapolis, IN) and stained with propidium iodide (Sigma-Aldrich, St. Louis, MO). DNA contents were measured with a flow cytometer (FACS; Becton Dickinson, San jose, CA).

### Western blot analysis

Whole cell lysates were prepared in lysis buffer containing 50 mM Tris PH 7.2, 1% Triton X-100, 0.5% sodium deoxycholate, 0.1% sodium dodecyl sulfate, 500 mM NaCl, and 10 nm MgCl_2_ with 10 µg/ml leupeptin, 10 µg/ml aprotinin, and 1 mM PMSF. The total proteins were resolved over 12% SDS-PAGE and transferred to nitrocellulose membranes (Immobilin-P; Millipore, Bedford, MA). The membranes were blocked with 5% nonfat milk at room temperature for 1 hour and then incubated with primary antibodies at 4°C overnight, followed by incubation with peroxidase-conjugated secondary antibodies for 2 hours at room temperature. Immunoreactive bands were detected and developed in ECL (enhanced chemiluminescence) system (Amersham Pharmacia Biotechnology, Uppsala, Sweden) with X-ray film. Antibodies against RPL6, cyclin E, CDK2, p16, p21, p27 were obtained from Santa Cruz Biotechnology (Santa Cruz, CA). Anti-β-actin was from Sigma (St. Louis, MO). Peroxidase-conjugated goat anti-mouse and goat anti-rabbit IgG were purchased from Boster (Wuhan, Hubei, China).

### Statistical Analysis

Statistical analyses were performed using SPSS statistical software (SPSS, Inc., Chicago, Illinois).Categorical variables were compared with chi-square test, the survival of the patients were evaluated by the Kaplan- Meier method, and the log-rank test was used to compare the differences. Mann-Whitney U test and Kruskal-Wallis H test were adopted for other data. Significance was defined as P<0.05.
